# P-407. Adherence to Clinical Pathways in the Management of Pediatric Skin and Soft Tissue Infections

**DOI:** 10.1093/ofid/ofaf695.624

**Published:** 2026-01-11

**Authors:** Harshini Kumaresan, Amanda Nedved, Frances Turcotte-Benedict, Brian R Lee, Rana E El Feghaly

**Affiliations:** University of Missouri-Kansas City, Kansas City, MO; Children's Mercy Kansas City; University of Missouri Kansas City School of Medicine, Lenexa, Kansas; Children's Mercy Kansas City; UMKC School of Medicine, Kansas City, Missouri; Children's Mercy Kansas City, Kansas City, Missouri; Children's Mercy Kansas City, Kansas City, Missouri

## Abstract

**Background:**

Skin and soft tissue infections (SSTIs) are a leading cause of pediatric emergency department (ED) and urgent care clinic (UCC) visits. While guidelines recommend limited diagnostic testing and short-course antibiotics for uncomplicated cases, practice often varies. Few studies have evaluated whether sociodemographic factors influence adherence to SSTI clinical pathways.

**Table 1:**
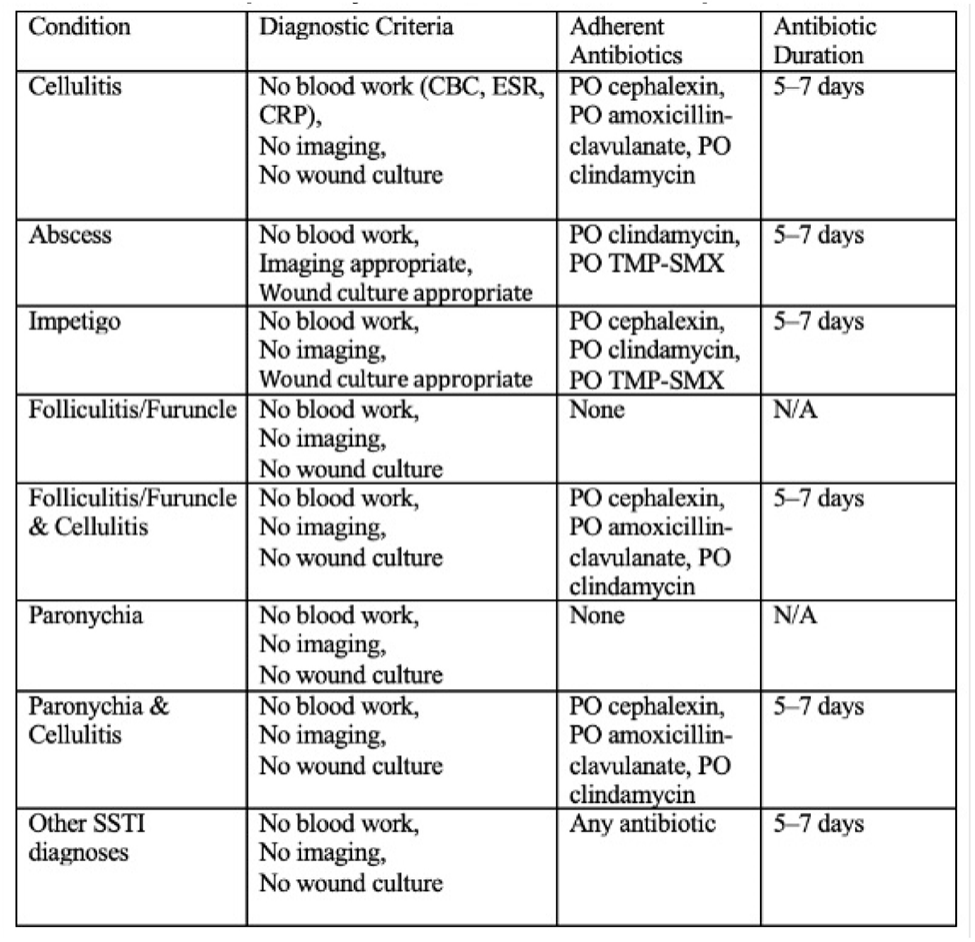
Clinical pathway adherence criteria for pediatric SSTIs

**Table 2:**
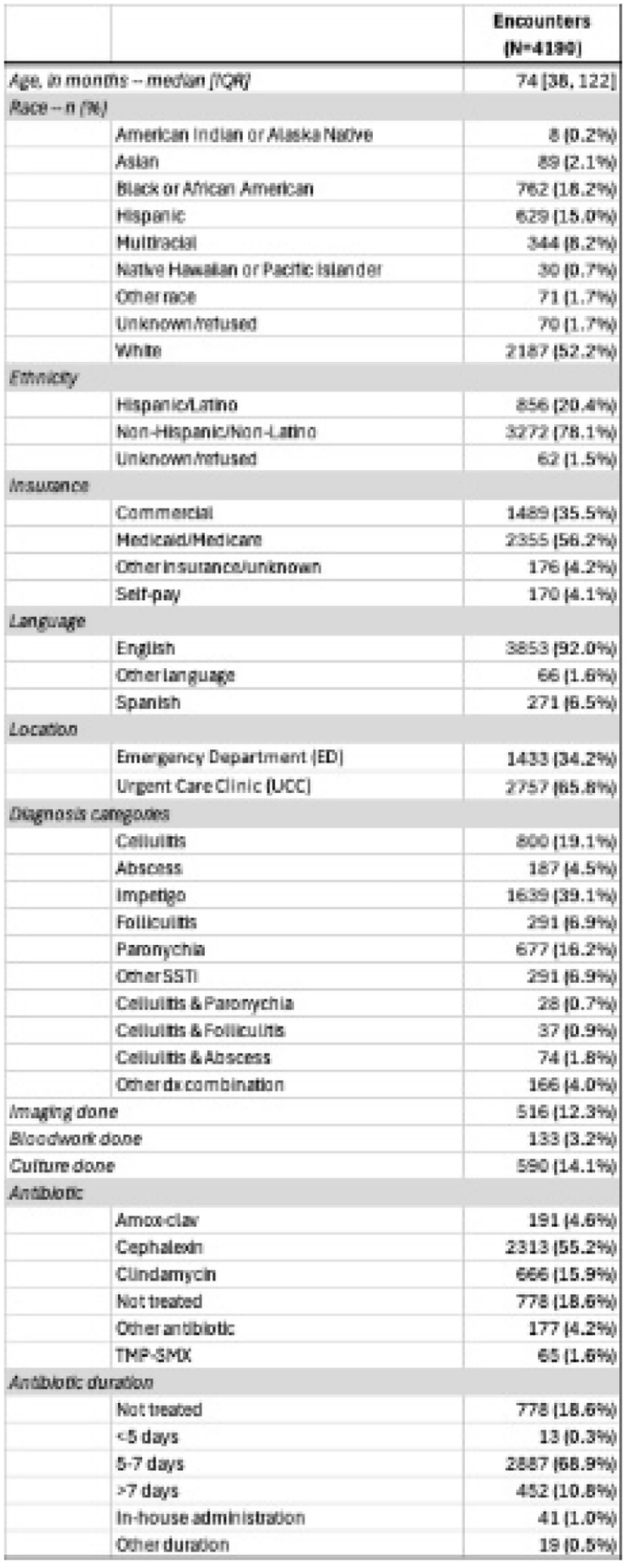
Demographics of our study population

**Methods:**

We retrospectively reviewed patients ages 60 days-19 years seen in Children’s Mercy Hospital (CMH) EDs or UCCs (July 2022-June 2024) with an SSTI ICD-10 diagnosis. Exclusions included hospitalization, immunocompromised, recent SSTI visits, and other infectious conditions. We defined overall adherence as no bloodwork, imaging, or wound culture unless indicated by national guidelines or our clinical pathway (CP), and adherence with antibiotic regimen (Table 1). We assessed appropriate antibiotic treatment selection and duration and return visit within 14 days. Bivariate analyses were used to compare proportions of CP adherence between select demographic factors (race, area-deprivation index [ADI], insurance, interpreter use).

**Table 3:**
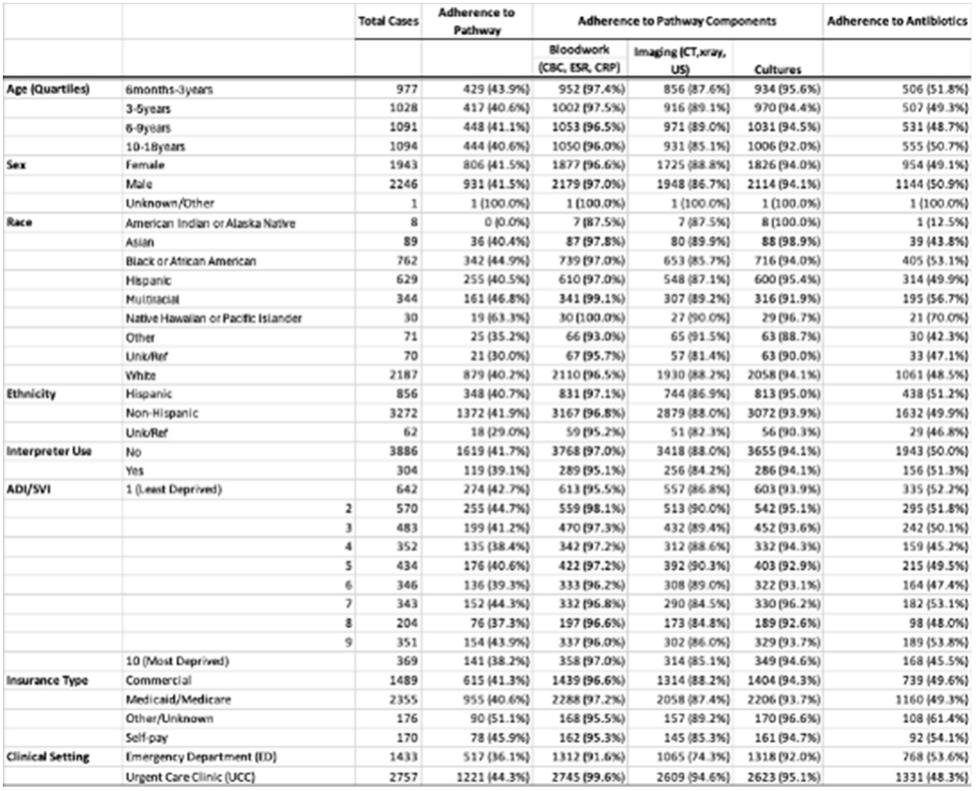
Adherence to clinical pathway

**Figure 1:**
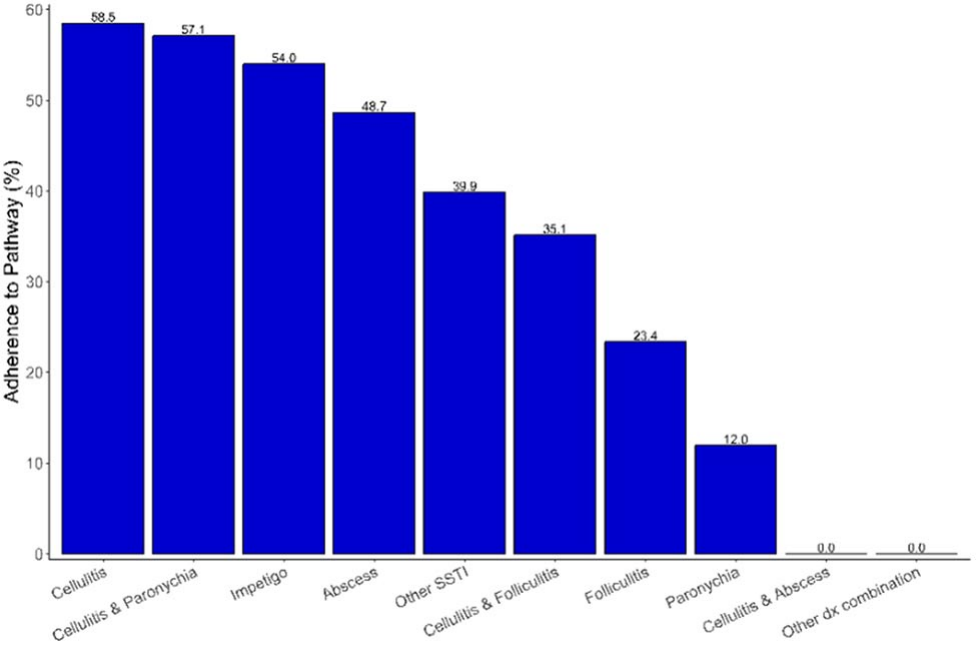
Adherence to clinical pathway by diagnosis

**Results:**

We identified 4,190 pediatric encounters for SSTIs with impetigo being the most common diagnosis (Table 2). The median age was 74 months (IQR 38–122); 18.2% were Black, 52% White, and 20.4% Hispanic. Adherence for bloodwork and imaging was lower in the ED compared to UCCs, but adherence to those was high for all sociodemographic groups (Table 3). Antibiotic adherence rates were highest among Black and multiracial children, and children 6 months–3 years (Table 3). Folliculitis and paronychia had the lowest adherence due to antibiotic overuse (Figure 1). Among patients with wound cultures (n=590), over 90% were positive, with MRSA more frequently identified in Black and Hispanic children. Fourteen-day return visit rates were lower among adherent patients (2.1% vs. 2.9%).

**Conclusion:**

Adherence to CP for diagnostic workup in pediatric SSTIs was consistently high, whereas antibiotic adherence varied by infection type, with lower adherence observed in folliculitis and paronychia. We did not observe significant differences in adherence based on race, ethnicity, language, insurance, and ADI.

**Disclosures:**

Amanda Nedved, M.D., Merck: Grant/Research Support Brian R. Lee, PhD, MPH, Merck: Grant/Research Support Rana E. El Feghaly, MD, MSCI, CPHQ, Merck (Any division): Grant/Research Support|Pfizer: Honoraria|Pfizer: Grant review panel

